# Microstructure and Corrosion Resistance of Laser-Welded Crossed Nitinol Wires

**DOI:** 10.3390/ma11050842

**Published:** 2018-05-18

**Authors:** Peng Dong, Runhua Yao, Zheng Yan, Zhifeng Yan, Wenxian Wang, Xiuli He, Jun Zhou

**Affiliations:** 1 College of Materials Science and Engineering, Taiyuan University of Technology, Taiyuan 030024, China; yrh_77@163.com (R.Y.); Z_Yan@tju.edu.cn (Z.Y.); wwx960@126.com (W.W.); 2 College of Materials Science and Engineering, Tianjin University, Tianjin 300072, China; 3 Department of Mechanical Engineering, Taiyuan Institute of Technology, Taiyuan 030008, China; hexl_ocean@163.com; 4 Department of Mechanical Engineering, Pennsylvania State University, Erie, PA 16563, USA

**Keywords:** nickel–titanium, laser welding, intermetallics, corrosion

## Abstract

Laser welding has been considered to be one of the most promising joining processes for Nitinol medical device manufacturing. Presently, there is still a limited understanding about how laser welding affects the microstructure and the resultant corrosion behaviors. This work aimed to reveal the microstructural factors that influence the corrosion resistance of laser-welded crossed Nitinol joints. The microstructures within various zones of the joints were characterized by using transmission electron microscopy (TEM), and the corrosion behaviors of the joints in 0.9% NaCl and Hank’s solutions were studied. The base metal exhibits a single austenite (B2) phase and the highest corrosion resistance. The phase constituent of the fusion zone is the coexistence of the B2 matrix and some precipitates (T_2_Ni, TiNi_3,_ and Ti_3_Ni_4_ particles), resulting in a slight decrease in corrosion resistance. The heat affected zone (HAZ) shows the austenite matrix but with the precipitation of R-phase, which considerably reduces the corrosion potential, making it the weakest zone.

## 1. Introduction

Because of the excellent biocompatibility, shape memory effect, and superelasticity, Nitinol is especially popular in the biomedical industry, where devices, such as stents, filters, and orthopedic implants, are manufactured [1,2,3,4,5]. Recently, there is an increasing clinical demand for the miniaturization of stents so that these can be surgically implanted into brain blood vessels, which are characterized by an extremely small diameter and highly tortuous extension [6,7,8]. However, it is challenging to prepare such fine and complex medical implants by laser cutting techniques, owing to the limitation of fine Nitinol tube production, processing precision, and the high cost. In this case, laser welding has been considered to be one of the most promising methods for the fabrication of the next generation of Nitinol vascular stents [5,8].

Because the welded Nitinol joints are intended to be used in physiological environment as implants, it is necessary, therefore, to understand how the welding process affects the microstructure and the resultant corrosion resistance of Nitinol, and to assess this property for the long-term stability and biocompatibility of implants [9]. Several works have been carried out to study the corrosion behavior of laser-welded Nitinol in the body environment. Hsu et al. [10] investigated the corrosion characteristics of CO_2_ laser-welded NiTi alloy thin sheet, with the results showing that NiTi joints exhibited excellent corrosion resistance in H_2_SO_4_ and HNO_3_ solutions. In contrast, the NiTi joints showed a decrease in corrosion resistance when tested in artificial saliva, where the corrosion rate and passive current density of the fusion zone were significantly higher than these of the parent material. This decrease was attributed to the presence of intermetallics in the weld zone. However, microstructure observations for these intermetallic particles were not reported in their work.

Interestingly, Yan et al. [11,12] reported that the NiTi joint showed a higher corrosion resistance than the base metal, both in NaCl and Hank’s solutions. These researchers suggested that the improvement of corrosion properties of the NiTi laser joint was related to the absence of carbides and increase of the Ti/Ni ratio and the smooth weld surface. Similarly, Man et al. [13] reported that laser surface melting can significantly improve the corrosion resistance of NiTi alloy in 3% NaCl solution. However, they explained that the improvement of corrosion resistance of the samples treated in argon was due to the presence of some new phases, such as Ti_2_Ni, TiNi_3_, and B19′, which were identified using XRD analysis. Recently, Chan et al. [14,15] performed some distinct works on the corrosion behavior of laser-welded NiTi alloy in Hank’s solution. Local electrochemical measurements showed a decrease in pitting potential and an increase in corrosion density of the fusion zone (FZ) and the heat-affected zone (HAZ), which indicated that welding reduced the corrosion resistance of Nitinol. Moreover, pits were identified in the HAZ near the FZ/HAZ boundary, which suggested that the coarse grain of the HAZ should be blamed for the corrosion resistance deterioration. In another study, Chan et al. [16] reported that post-weld heat-treatment (PWHT) can significantly improve the pitting corrosion resistance. This increase in the corrosion resistance behavior was attributed to the Ni_4_Ti_3_ precipitation, instead of the grain size factor.

From the literature, it can be observed that the results on corrosion resistance of the laser-welded NiTi alloy are contradictory, and the microstructural origins of corrosion behavior are not well-known. Therefore, it is important to study the microstructure in detail, such that the corrosion behavior can be better understood and controlled. In this work, the detailed microstructure and corrosion behavior in NaCl and Hank’s solutions of laser-welded NiTi joints were investigated. Finally, the microstructural factors that influence corrosion susceptibility of laser-welded NiTi joint were discussed.

## 2. Materials and Methods

### 2.1. Materials and Laser-Welding

Starting materials were commercial super-elastic NiTi alloy (Nitinol, Ti–55.8 wt % Ni, Confluent Medical, Fremont, CA, USA) wires with a nominal diameter of 0.01 inch (about 254 μm). Before welding, surface oxides and contamination of the wires were removed by pickling (HF:HNO_3_:H_2_O = 1:4:5) for 20 s, and followed by ultrasonic cleaning within acetone solution for 5 min. The wires were then rinsed several times with deionized water and dried by airflow.

**Figure 1:**
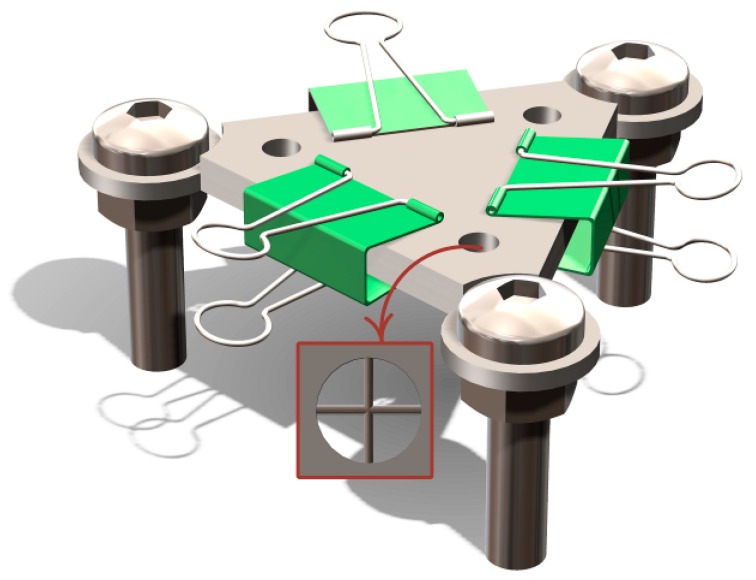
Schematic of the welding fixture.

Figure 1 shows the design drawing of the welding fixture being used to position the wires at 90° to one another in the holes. Binder clips were used to give a downward force, which ensures close contact between wires. A pulsed Nd:YAG laser system (W100B, Han’s Laser, Shenzhen, China) with a 1064 nm wavelength, 150 μm nominal spot diameter, and a Gaussian spatial profile was used to produce wire-to-wire crossed joints. The laser system was equipped with a stereomicroscope (Shanghai optical instrument factory, Shanghai, China), allowing for accurate adjustment of the laser at the intersection of the crossed wires. Single pulse laser welding was performed under Ar shielding (5 L·min^−1^), with a power of 300 W, pulse duration of 0.2 ms, and frequency of 1 Hz. Additionally, the defocus distance was set at 0.

### 2.2. Electrochemical Tests

Corrosion behavior of the Nitinol joint was evaluated, for both the entire joint and for the local zones (FZ, HAZ and BM), using electrochemical tests in a stroke-physiological saline solution (0.9% NaCl) and simulated body fluid (Hank’s solution: NaCl 8 g/L, Na_2_HPO_4_ 0.0475 g/L, NaHCO_3_ 0.35 g/L, KCl 0.4 g/L, KH_2_PO_4_ 0.06 g/L, MgCl_2_-6H_2_O 0.10 g/L, MgSO_4_-7H_2_O 0.10 g/L, CaCl_2_ 0.18 g/L, glucose 1 g/L, pH 7.4). The electrochemical cell was a standard three-electrode system, where a saturated calomel electrode (SCE) was used as the reference electrode, and graphite rods acted as the counter electrode.

All samples used in corrosion tests were mechanically ground using a SiC paper and later polished with a 1-μm diamond paste. After cleaning and rinsing, as described in Section 2.1., the samples were immersed in solution for 30 min to stabilize the open-circuit potential (OCP). Next, potentiodynamic tests were immediately performed using an electrochemical workstation (CS310; CorrTest, Wuhan, China). A scan rate of 0.5 mV/s was used in the range of −1–1.5 V with respect to OCP for all samples. The solution was maintained at 37 °C using a water bath during the tests. Surface morphology observations after corrosion tests were carried out using a field-emission scanning electron microscope (FESEM; MIRA3 LM, TESCAN, Kohoutovice, Czech).

### 2.3. Microstructure Characterization

The microstructures of Nitinol joints were examined using an optical microscopy (OM; Leica DM4, Wetzlar, Germany) and a transmission electron microscopy (TEM; JEOL 2100F, Tokyo, Japan). Before OM observation, the samples were prepared using standard metallographic procedures. To ensure accuracy, a focused ion beam (FIB) workstation (LYRA3, TESCAN) was utilized to prepare TEM foil samples by means of the lifting out technique. The TEM observations were performed at an acceleration voltage of 200 kV.

## 3. Results

### 3.1. Microstructure Analysis

**Figure 2:**
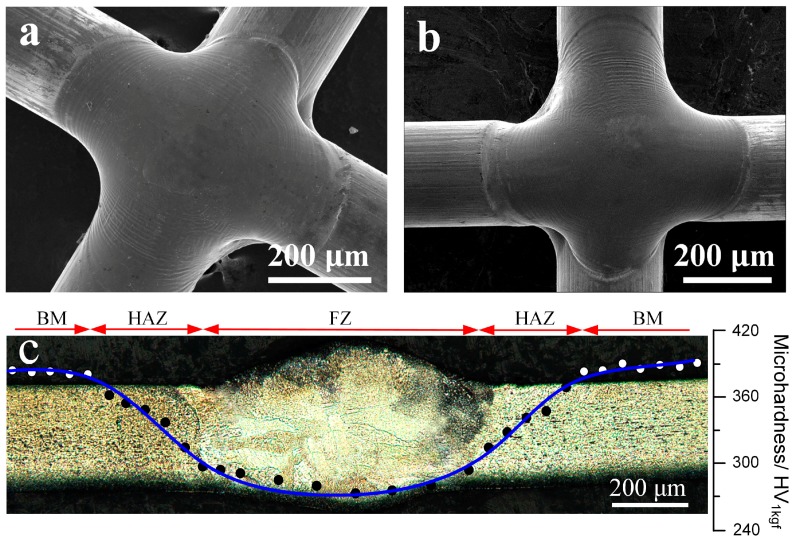
Scanning electron microscope (SEM) images of the crossed joint for the (**a**) front and (**b**) back surfaces, and overall observation of the cross-section with hardness distribution (**c**).

Figure 2a,b shows the SEM observations of the joint morphologies. Clearly, laser-welding could produce a defect-free joint with a smooth surface. In the case of medical implants, such a smooth surface could minimize damage to organs and tissues during implantation and operation [9]. Figure 2c shows the overall observation of the joint cross-section and the corresponding microhardness profile through the mid-thickness. Laser welding resulted in a U-shaped profile and a lower hardness (~270 HV_1kgf_) region compared to that of the base metal (~400 HV_1kgf_), which can be seen clearly in the center zone of the joint. A similar U-shaped hardness profile of the Nitinol laser joint has been reported by Tam et al. [17]. Based on the hardness distribution, three microstructural zones, the fusion zone (FZ), heat-affected zone (HAZ), and base metal (BM), can be identified.

**Figure 3:**
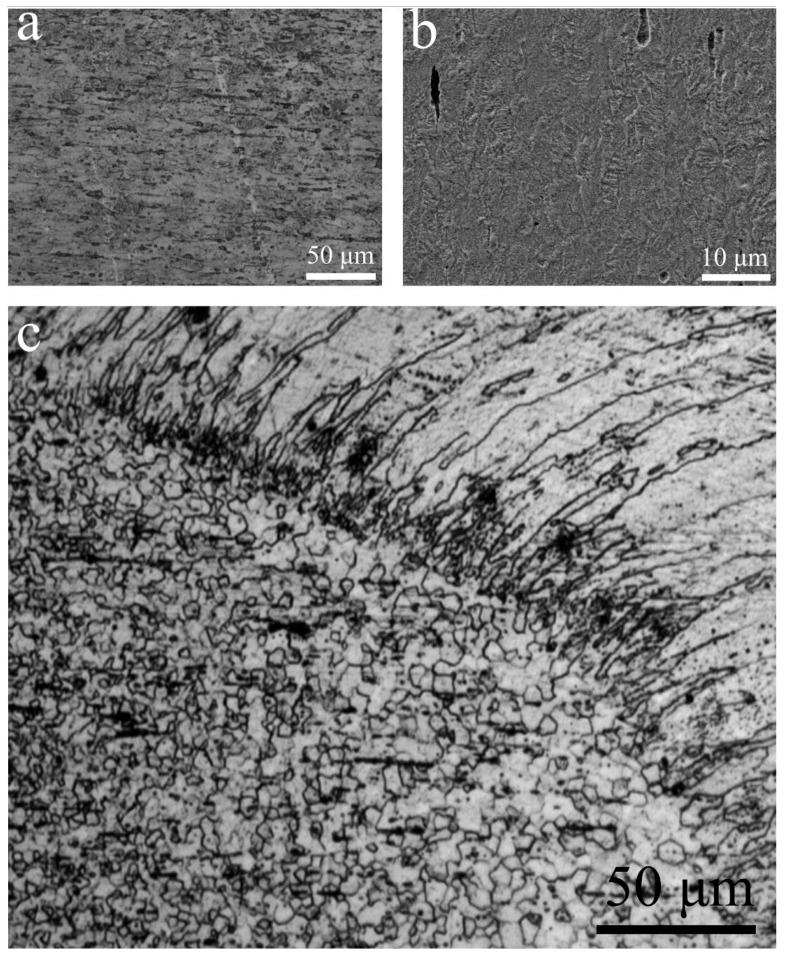
Grain structure of the base metal (**a**,**b**) and the transition zone (**c**).

Grain structure observations of the joint are presented in Figure 3. BM showed a fine unidirectional structure with indiscernible grain boundaries, which result from the cold drawing process (Figure 3a). Despite FESEM being set at a high magnification (Figure 3b), grain boundaries were hardly observed. HAZ near the fusion boundary exhibited equiaxed grains with an average size of about 7 μm, and FZ showed coarse columnar dendrites with an average width of about 20 μm (Figure 3c). It is quite common that the observed directional grains of the FZ near the fusion boundary are the results of epitaxial solidification and competitive growth. In this case, the cold hardening effects of the wire would be eliminated in FZ, resulting in the minimum hardness level. For HAZ, heat conduction into the wire would produce a gradient effect of recrystallization and growth of original grains, due to the high interior energy caused by cold working. This then leads to gradient elimination of previous cold working, resulting in the observed softening within the HAZ.

**Figure 4:**
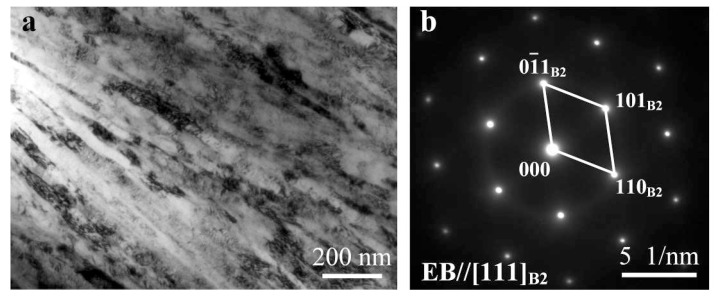
Transmission electron microscopy (TEM) image (**a**) and the corresponding selected area diffraction pattern (SADP) (**b**) of base metal (BM).

Figure 4 shows a TEM image and the corresponding selected area diffraction pattern (SADP) of BM, where grains are preferentially oriented along a specific direction. The length of the elongated grains exceeds 1.5 μm, while the width is about 40 nm, as shown in Figure 4a. Consequently, grain boundaries are not able to be distinguished in OM. The SADP (Figure 4b) result verifies that the BM is a single B2 phase structure.

**Figure 5:**
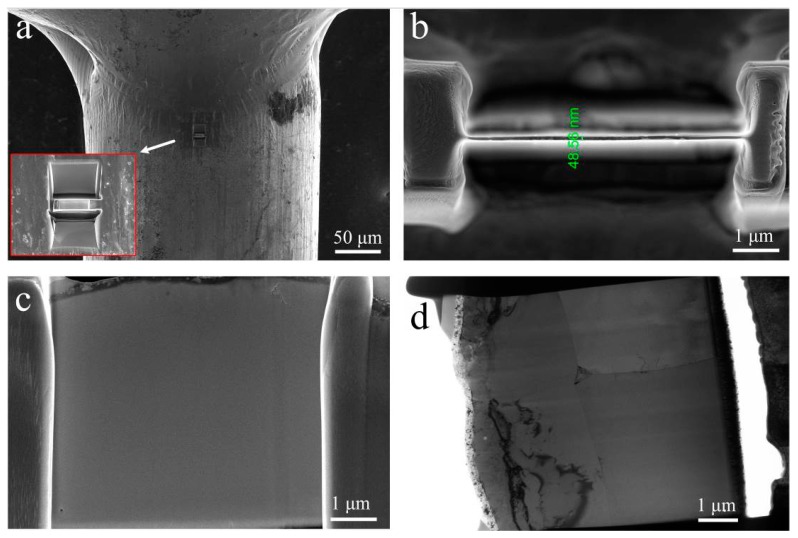
TEM foil removed from the heat-affected zone (HAZ) close to the fusion boundary using focused ion beam (FIB) (**a**) with a uniform thickness of ~50 nm (**b**), and overall examinations of foil using field-emission scanning electron microscope (FESEM) (**c**) and TEM (**d**).

Figure 5a shows that the FIB etching location of the HAZ was near the fusion boundary. The TEM foil was finally thinned to a uniform thickness of about 50 nm (Figure 5b). The overall observations of the foil using FESEM and TEM are presented in Figure 5c,d, which did not show any apparent sign of precipitates in the FZ.

**Figure 6:**
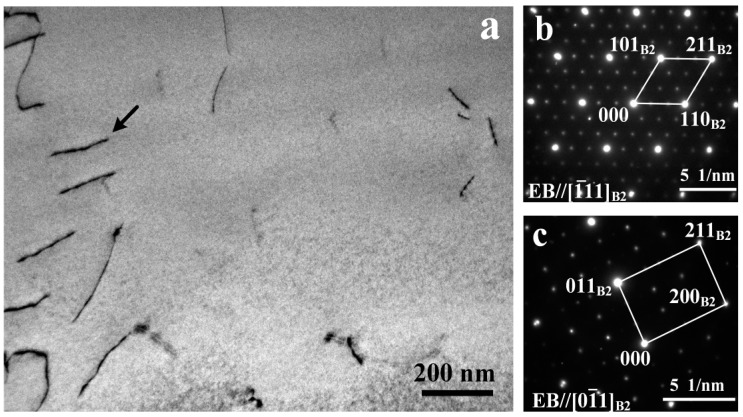
TEM image of HAZ (**a**) and the corresponding SADP under [−1 1 1]_B2_ (**b**), and [0 −1 1]_B2_ (**c**) zone axis.

Figure 6a shows the TEM micrograph at high magnification taken from the HAZ. Several dislocations were clearly observed, and dark contrast arising from extremely fine particles was found. The shape of fine precipitates is not well-defined as they are extremely fine (~10 nm). Furthermore, SAD was performed under [−1 1 1]_B2_ and [0 −1 1]_B2_ zone axis, as shown in Figure 6b,c. These patterns were characterized with B2 diffraction spots and one-third reflections, which confirmed the R-phase identity according to the previous studies [18,19].

**Figure 7:**
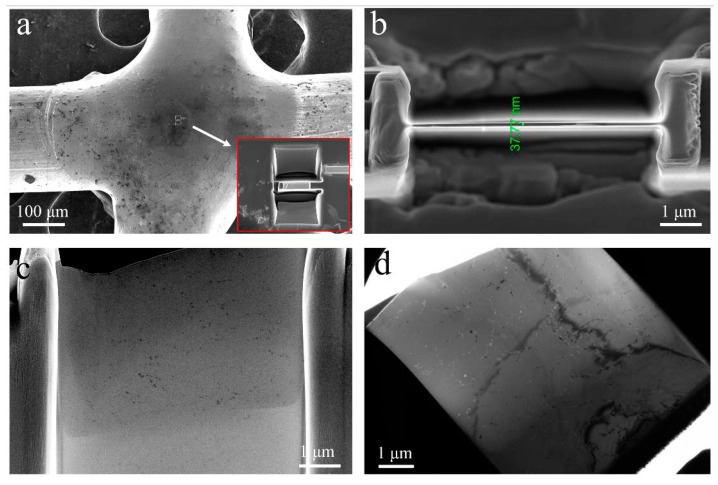
TEM foil removed from the fusion zone (FZ) center using FIB (**a**) with a uniform thickness of ~40 nm (**b**), and overall examinations of foil using SEM (**c**) and TEM (**d**).

The preparation of TEM foil of FZ was located at the center, as shown in Figure 7a. The foil was finally thinned to a uniform thickness of ~40 nm (Figure 7b). The overall observations of the foil using FESEM and TEM are presented in Figure 7c,d, with both showing the presence of fine second phase particles.

**Figure 8:**
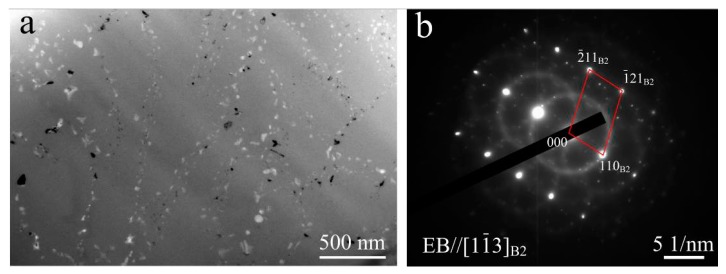
TEM image of FZ (**a**) and the corresponding SADP under [1 −1 3]_B2_ zone axis (**b**), showing B2 matrix with complex precipitations.

**Figure 9:**
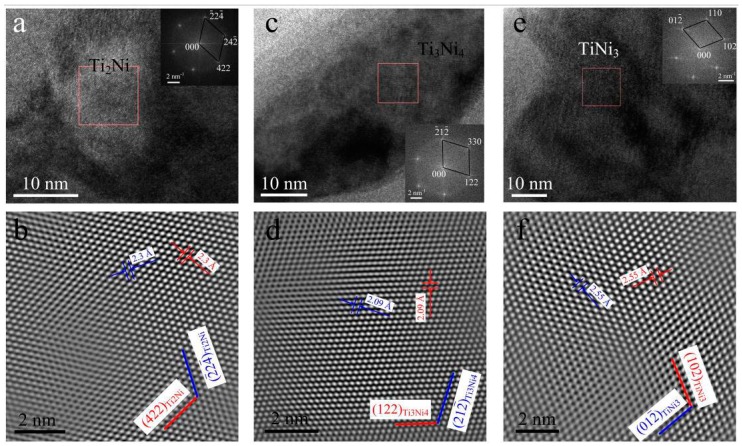
Identification of precipitates within the FZ via HRTEM images inserted with local fast fourier transform (FFT) patterns (**a**,**c**,**e**) and the corresponding inverse FFT images (**b**,**d**,**f**).

Figure 8a presents the TEM micrograph at high magnification taken from FZ, which shows a large number of nanoscale particles with white, gray white, and dark kinds of contrast. Selected area diffraction for the entire region was performed and the result revealed that the phase constituent of FZ is the B2 phase, but with precipitates (as shown in Figure 8b). However, it is difficult to determine the exact composition of the precipitates. In this case, HRTEM observations for precipitates were carried out and the results are shown in Figure 9, which verified that the gray white, white, and dark particles are Ti_2_Ni, Ti_3_Ni_4,_ and TiNi_3_ compounds, respectively.

### 3.2. Corrosion Behavior of Nitinol Joints

**Figure 10:**
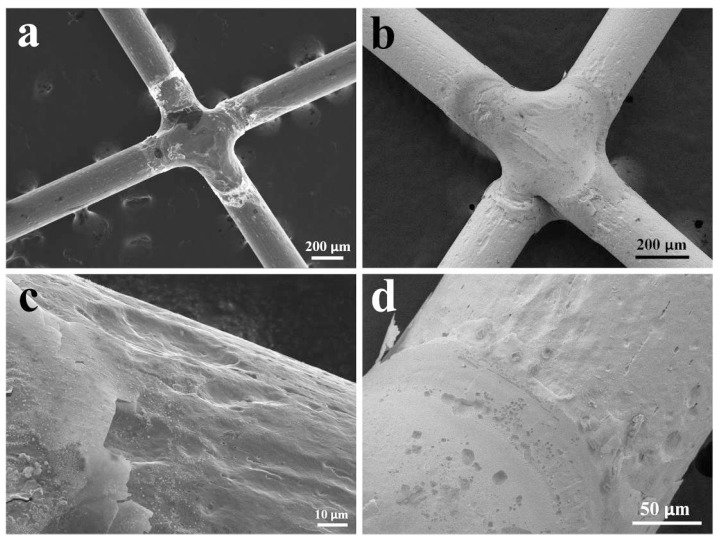
FESEM observation of Nitinol joint after corrosion test in 0.9% NaCl solution (**a**,**c**) and Hank’s solution (**b**,**d**).

Figure 10 shows FESEM observations of the surface morphologies of Nitinol joints after corrosion tests in 0.9% NaCl and Hank’s solutions, providing information for the corrosion susceptibility of microstructural zones. It can be concluded from Figure 10a,b that the most severe damage was located at the HAZ (adjacent to the fusion line) in both solutions, and the BM was barely affected by the corrosion. High magnification observations (Figure 10c,d) indicate that corrosion damage primarily originated from large pits. In addition, the Nitinol joint is more susceptible to the 0.9% NaCl solution compared with Hank’s solution.

**Figure 11:**
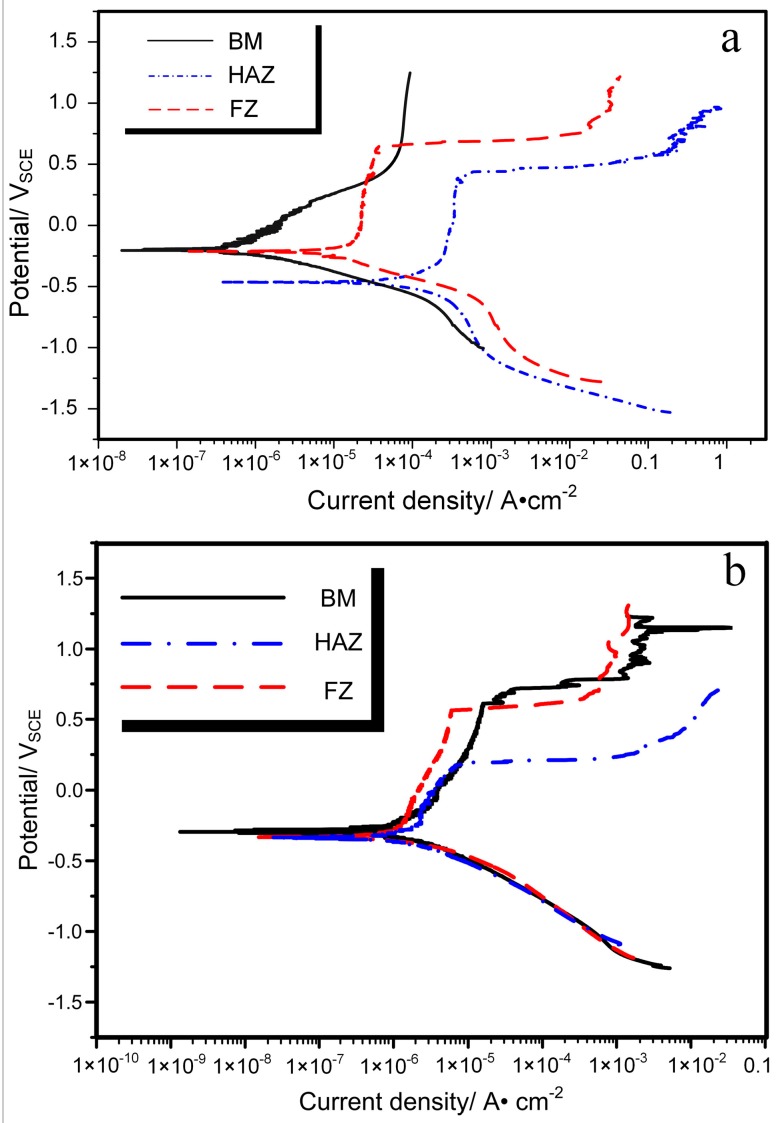
Polarization curves of the three microstructural zones (FZ, HAZ, and BM) conducted in 0.9% NaCl solution (**a**) and Hank’s solution (**b**).

To examine the local corrosion behavior, potentiodynamic tests for the three microstructural zones (FZ, HAZ, and BM) were conducted in two solutions, and the polarization curves are shown in Figure 11. The corrosion potential (*E_corr_*), pitting potential (*E_pit_*), corrosion current density (*I_corr_*), and corrosion rate (*R_corr_*) are extracted from these curves, as shown in Table 1 and Table 2. It is well-known that *E_corr_* represents the thermodynamic stability or corrosion resistance, which means that the higher the value of *E_corr_* is, the more resistant it is to corrosion. *E_pit_* refers to the critical potential above which the passive film breaks down and pits originate on the free surface of the specimen. Thus, a higher value of *E_pit_* indicates a larger passivation range and a higher corrosion resistance. Moreover, the values of *I_corr_* and *R_corr_* illustrate how fast the material will be lost during the corrosion process. Hence, the high value of the two parameters means the sufficient corrosion kinetics are satisfied.

It can be observed from Table 1 that the HAZ shows the lowest value of *E_corr_* and *E_pit_* and the highest value of *I_corr_* and *R_corr_* in a 0.9% NaCl solution. Such parameters show similar trends in Hank’s solution (Table 2). However, the difference in the values among different microstructural zones decreases, except for *E_pit_*. Both the surface morphology and electrochemical parameters indicate that HAZ is the weakest zone of corrosion.

## 4. Discussion

The difference in corrosion susceptibility of Nitinol joint must be considered to a result of the presence of a heterogeneous microstructure caused by welding. It is generally accepted that the corrosion resistance of a material primarily depends on the level of corrosion potential and the stability of passive layer, as well as the surface finish [11,12,13,14,15,16].

The high corrosion resistance of Nitinol BM can be attributed to single B2 phases, which exhibit high thermodynamic stability or high corrosion potential. As a result, BM preferentially acts as a cathode in a galvanic couple. In addition, the excellent corrosion resistance also benefits from its low corrosion current density, which shows that the passive film is stable.

**Figure 12:**
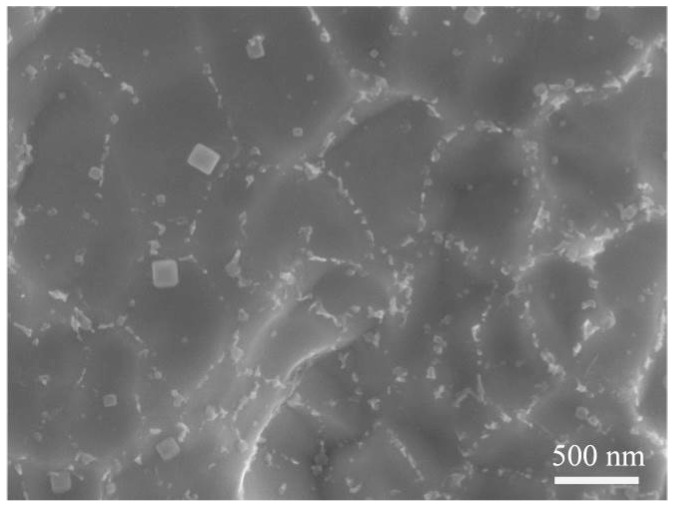
FESEM image taken from FZ after corrosion test.

Microstructural analysis revealed that laser-welding resulted in the precipitation of intermetallic particles in the fusion zone. Many researchers [11,12,13] have reported that these intermetallic particles could provide initiation sites for the breakdown of passive films and then act as preferential sites for pit initiation in an equiatomic NiTi alloy. This is consistent with our findings. Figure 12 presents the FESEM image taken from the FZ after the corrosion test, which shows a large number of particles. However, due to the disturbance of residual Na^+^ and Cl^−1^, as well as the limitation of energy disperse spectroscopy (EDS) examination, it is difficult to obtain the exact compositions of these particles. Interestingly, it is observed that the morphology, size, and distribution of the particles are extremely similar to the precipitates identified via TEM (Figure 8a). This finding means that the precipitates (such as Ti_2_Ni, TiNi_3,_ and Ti_3_Ni_4_) were left over, while the B2 matrix was dissolved during the corrosion process. This indicates that these precipitates may have a higher corrosion potential than the B2 phase. The presence of these precipitates leads to the increase of corrosion potential in FZ, thereby resulting in the corrosion resistance improvement. However, the dissolution of matrix surrounding the precipitates is inevitable due to the galvanic couple effect.

**Figure 13:**
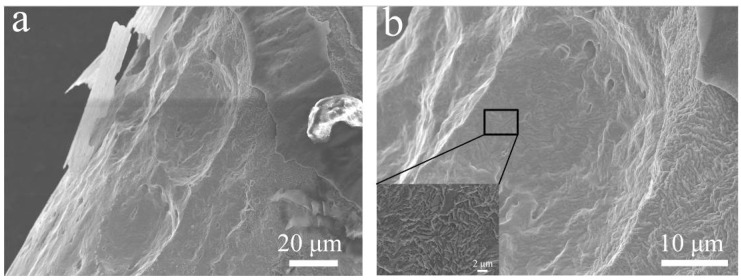
FESEM micrographs taken from the HAZ after test show the broken passive layer (**a**) and the slice-like features (**b**).

HAZ has been demonstrated to be the weakest zone for corrosion. Chan et al. [14] also reported similar results of laser-welded NiTi alloy. FESEM micrograph (Figure 13) taken from the HAZ after the test reveals the broken passive layer and the slice-like features. Considering that the grain size in the HAZ is far larger than the slices, it is reasonable to believe that the corrosion was intragranular. Based on the electrochemical parameters of this zone, the severely localized corrosion in the HAZ can be explained by its low value of *E_corr_* and *E_pit_*, as well as the high level of *I_corr_*. Microstructural analysis for the HAZ reveals the recrystallized grains, and coexistence of B2 and R phases. Chan et al. [14,15] suggested that the coarse recrystallized grains lead to the significant deterioration of corrosion resistance. Their explanation was based on the findings from Liu and Duh [20], who reported that the localized corrosion resistance of NiTi in NaCl solution increases with decreasing grain size. However, our study on grain structure of NiTi joint showed that the grains in the FZ were coarser than that in the HAZ, and corrosion tests showed that the FZ was more resistant to corrosion than the HAZ. These results indicated that the grain size is not the decisive factor of corrosion resistance for the Nitinol joint. In this case, the precipitation of R-phase in the HAZ should be to blame for the corrosion deterioration.

Additionally, it is a common for Ti alloys to form a calcium–phosphorous layer on their outermost surface naturally after exposure to a simulated body fluid [21]. This Ca–P layer may serve as a further barrier against ion diffusion. In fact, it has been demonstrated that the formation of the Ca–P layer is controlled by the existence of titanium oxide [22]. The surface concentration of calcium and phosphorous for the different microstructural zones of the joint was almost the same for the BM, HAZ, and FZ. As a result, the differences in electrochemical parameters (Table 2) of different microstructural zones in Hank’s solution is very small.

## 5. Conclusions

Laser-welding significantly changes the microstructure and corrosion susceptibility of Nitinol. The base metal is characterized by unidirectional grains, is composed of a single B2 phase, and exhibits the highest corrosion resistance in both corrosive media. After welding, the fusion zone presents coexistence of the B2 matrix and several intermetallics (T_2_Ni, TiNi_3,_ and Ti_3_Ni_4_), which results in a decrease of *E_corr_* and *E_pit_*, and an increase of *I_corr_* and *R_corr_*. The formation of R-phase in the HAZ significantly reduces the corrosion resistance, with low *E_corr_* and *E_pit_*, and relatively high *I_corr_* and *R_corr_*, making it the weakest zone.

## Figures and Tables

**Table 1:** Electrochemical parameters of the local region of Nitinol joint in 0.9% NaCl solution.

Sample	*E_corr_*, mV_SCE_	*E_pit_*, mV_SCE_	*I_corr_*, μA·cm^−2^	*R_corr_*, μm·Year^−1^
BM	−198	-	0.6	4.0
FZ	−210	641	28.4	18.6
HAZ	−468	404	111.3	739.8

**Table 2:** Electrochemical parameters of the local region of Nitinol joint in Hank’s solution.

Sample	*E_corr_*, mV_SCE_	*E_pit_*, mV_SCE_	*I_corr_*, μA·cm^−2^	*R_corr_*, μm·Year^−1^
BM	−302	703	0.7	4.5
FZ	−328	568	1.2	7.6
HAZ	−336	198	2.4	15.8
